# Revealing the biological features of the axolotl pancreas as a new research model

**DOI:** 10.3389/fcell.2025.1531903

**Published:** 2025-01-31

**Authors:** Hui Ma, Guangcong Peng, Yan Hu, Binbin Lu, Yiying Zheng, Yingxian Wu, Weimin Feng, Yu Shi, Xiangyu Pan, Li Song, Ina Stützer, Yanmei Liu, Jifeng Fei

**Affiliations:** ^1^ Department of Pathology, Guangdong Provincial People’s Hospital (Guangdong Academy of Medical Sciences), Southern Medical University, Guangzhou, Guangdong, China; ^2^ BGI Research, Qingdao, China; ^3^ School of Basic Medical Sciences, Southern Medical University, Guangzhou, China; ^4^ Key Laboratory of Brain, Cognition and Education Sciences, Guangdong Key Laboratory of Mental Health and Cognitive Science, Ministry of Education, Institute for Brain Research and Rehabilitation, South China Normal University, Guangzhou, China; ^5^ The Innovation Centre of Ministry of Education for Development and Diseases, School of Medicine, South China University of Technology, Guangzhou, China; ^6^ Guangdong Provincial Key Laboratory of Medical Immunology and Molecular Diagnostics, Guangdong Medical University, Dongguan, China; ^7^ Deutsche Forschungsgemeinschaft (DFG)-Center for Regenerative Therapies Dresden, Technische Universität Dresden, Dresden, Germany

**Keywords:** axolotl, diabetes, glucose metabolism, pancreas development, *Pdx1* mutation

## Abstract

**Introduction:**

The pancreas plays a crucial role in digestion and blood glucose regulation. Current animal models, primarily mice and zebrafish, have limited the exploration of pancreatic biology from an evolutionary-developmental perspective. Tetrapod vertebrate axolotl (*Ambystoma mexicanum*) serves as a valuable model in developmental, regenerative, and evolutionary biology. However, the fundamental biology of the axolotl pancreas remains underexplored. This study aims to characterize the unique developmental, functional, and evolutionary features of the axolotl pancreas to expand the understanding of pancreatic biology.

**Methods:**

We conducted morphological, histological, and transcriptomic analyses to investigate the axolotl pancreas. Pancreatic development was observed using *in situ* hybridization and immunostaining for key pancreatic markers. RNA sequencing was performed to profile global gene expression during larva and adult stages. And differential gene expression analysis was used to characterize the conserved and unique gene patterns in the axolotl pancreas. Functional assays, including glucose tolerance tests and insulin tolerance tests, were optimized for individual axolotls. To assess pancreatic gene function, *Pdx1* mutants were generated using CRISPR/Cas9-mediated gene editing, and their effects on pancreatic morphology, endocrine cell populations, and glucose homeostasis were analyzed.

**Results:**

The axolotl pancreas contains all known pancreatic cell types and develops from dorsal and ventral buds. Both of buds contribute to exocrine and endocrine glands. The dorsal bud produces the major endocrine cell types, while the ventral bud generates α and δ cells, but not β cells. Differential gene expression analysis indicated a transition in global gene expression from pancreatic cell fate commitment and the cell cycle to glucose response, hormone synthesis, and secretion, following the development progression. Notably, the adult axolotl pancreas exhibits slower metabolic activity compared to mammals, as evidenced by the results of GTT and ITT. The mutation of *Pdx1* resulted in hyperglycemia and a significant reduction in pancreatic cell mass, including a complete loss of endocrine cells, although it did not lead to a lethal phenotype.

**Discussion:**

This study examines the axolotl pancreas, highlighting the conservation of pancreatic development. Our study highlights the unique features of the axolotl pancreas and broadens the scope of animal models available for pancreatic evolution and disease research.

## 1 Introduction

The pancreas is an important organ in the mammalian digestive system, composed of exocrine and endocrine cells that release enzymes for digestion and hormones to regulate blood glucose metabolism. Clustering of the endocrine cells forms the islets of Langerhans in mammals, which consist of five different endocrine cell types, Insulin (INS)-secreting β cells, Glucagon (GCG)-producing α cells, Somatostatin (SST)-secreting δ cells, Pancreatic polypeptides (PPY)-producing PP cells and ε cells that secrete Ghrelin (GHRL) (Lewis and Mao, 2023; Steiner et al., 2010; Bonner-Weir et al., 2015). These endocrine cells regulate each other’s secretion and work synergistically to maintain blood glucose level (Röder et al., 2016). Diabetes mellitus, characterized by hyperglycaemia due to absolute or relative insulin insufficiency arising from β cell impairments in human (Cole and Florez, 2020; Ghila et al., 2022), is one of the most significant challenges in public health (Hossain et al., 2024). Investigating the mechanisms of pancreatic development and regeneration using model organisms can enhance our understanding of how endocrine cells are generated and regenerated, providing insights into the fundamental principles of organ development and potential regenerative therapies for pancreatic diseases.

Currently, animal models such as mice and zebrafish are the most commonly used organisms for studying the development and regeneration of pancreatic β cells (Aguayo-Mazzucato and Bonner-Weir, 2018; Yang et al., 2020). The pancreas of rodents and zebrafish exhibits significant similarity in cell type composition and gene expression programs during development and homeostasis (Slack, 1995). However, they also exhibit certain variations that can confer advantage or disadvantage for pancreas research (Mi et al., 2024; Weng et al., 2019). While rodents are evolutionarily closer to humans, resulting in similarities in pancreatic structure and the state of exocrine and endocrine cells, they present limitations in the context of pancreatic regeneration. Notably, adult mice have a relatively low β cell replication rate and are unable to restore β cell mass under type I and type II diabetic conditions (Rankin and Kushner, 2009; Sasaki et al., 2021; Teta et al., 2005). In contrast, zebrafish exhibit a higher capacity for β cell regeneration, providing advantages over mammalian models for studies on pancreatic regeneration (Mi et al., 2024; Moss et al., 2009). Moreover, the transparency and small size of zebrafish make them ideal for live imaging of the pancreas and drug screening, including the identification of compounds that promote β cell regeneration using genetically modified strains (Li et al., 2021; Teame et al., 2019). However, the small body size of zebrafish presents challenges for examining physiological parameters of glucose metabolism, such as glucose tolerance test (GTT) and insulin tolerance test (ITT) (Kimmel and Meyer, 2016; Yang et al., 2020). Consequently, exploring new animal models positioned at crucial evolutionary nodes and exhibiting unique biological features will enrich the resources available for pancreatic research and provide additional insights into pancreatic development, regeneration, and evolution.

The axolotl, a tetrapod vertebrate, possesses remarkable regenerative capacities (Vieira et al., 2020). Previous studies have demonstrated that many organs in axolotls, including limbs, tails, brains, spinal cords, hearts and lenses, can regenerate following injury (Bryant et al., 2017; Cano-Martínez et al., 2010; Cura Costa et al., 2021; Echeverri and Tanaka, 2002; Lust et al., 2022; Suetsugu-Maki et al., 2012; Wei et al., 2022). From an evolutionary perspective, axolotls belong to amphibians and are classified between fish and rodents (Lu, 2023). Despite extensive research on canonical organs in axolotls, the pancreas has been poorly characterized, limiting its application in pancreatic biology. Recently, Sørensen and colleagues identified the presence of islets in the axolotl pancreas using Hematoxylin-Eosin (H&E) staining and immunohistochemistry with an insulin antibody on tissue sections. They also demonstrated streptozotocin-induced hyperglycemia, detected via intraperitoneal GTT (Sørensen et al., 2022). However, little is known about the complete cellular composition, developmental features, gene expression, and genetic regulation of pancreatic function in axolotls as a potential model for diabetes mellitus.

In this study, we reported that the axolotl pancreas comprises ducts, acinar cells and islets, which contain α, β, δ, and ε cells. Notably, we observed that the PP cells, which secrete PPY, co-expressed the typical α cell marker GCG. Developmentally, we found that a significant portion of the pancreas originates from the dorsal bud, while the uncinate process, derived from the ventral bud, contains α and δ cells but no β cells. At the gene expression level, we noted strong conservation of gene regulation in axolotl pancreas during development and homeostasis, although it exhibited slower metabolic activity compared to mammals. Moreover, following the mutation of Pancreatic and duodenal homeobox 1 (*Pdx1*), the mutant axolotls survived but exhibited a hyperglycemic phenotype, deficiencies in the exocrine pancreas, and varying degrees of loss of endocrine cells. Additionally, intraperitoneal GTT and ITT revealed a delayed response to glucose and insulin in the axolotl pancreas, consistent with our gene expression analysis and suggesting a slower rate of glucose metabolism. Overall, our study characterizes the fundamental biological features of the axolotl pancreas and lays the groundwork for using axolotl as a model for pancreatic research.

## 2 Materials and methods

### 2.1 Animal husbandry

The axolotl (*Ambystoma mexicanum*) *d/d* strain used in this study was maintained in fresh water, with individuals housed separately and fed daily. Prior to surgical procedures, axolotls were anaesthetized using a 0.01% solution of ethyl-p-aminobenzoate (Benzocaine; Sigma). All animal experiments were carried out according to the guidelines of the ethics committee of Guangdong Provincial People’s Hospital (approval number KY-Q-2022-395-02).

### 2.2 Generation of *Pdx1* mutant axolotls

*Pdx1* mutant axolotls were generated using CRISPR/Cas9 technology, following established protocols. The *Pdx1* guide RNA (gRNA) sequence (5′-AAG​GAG​GAG​GAC​AAG​AAG​CG-3′) was synthesized by GenScript. A mixture containing 0.4 μg/μL *Pdx1* gRNA, 0.5 μg/μL Cas9 protein, and 1× Cas9 buffer was injected into single-cell stage embryos from the *d/d* strain, as previously described (Fei et al., 2018). The injected embryos developed into juveniles, which were subsequently genotyped to identify the F_0_ transgenic animals. Genomic DNA was extracted from axolotl tail snips using a solution containing 50 mM NaOH and 1 M Tris-HCl. The target locus of the *Pdx1* gRNA was PCR amplified using primers Pdx1-KOfw1 (5′-act​tga​cga​gct​gtt​cgc​cca​ttt​c-3′) and Pdx1-KOre2 (5′-tga​gca​tgt​cct​tgc​cag​ggt​ccc-3′). The PCR products were then subjected to Sanger sequencing (Tsingke) for alignment with the original *Pdx1* sequence. F_0_ transgenic axolotls were further bred to generate F_1_ offspring with various genotypes (Supplementary Figure S1).

### 2.3 Immunofluorescence

Samples were fixed in 3.7% formaldehyde freshly prepared in MEM buffer (MEMFA) overnight at 4°C. After dehydration in 30% sucrose, the samples were embedded in OCT (Sakura, 4,583). Cryosections of 10 µm were then collected for immunohistochemical analysis as previously described (Fei et al., 2017). The sections were blocked with a blocking buffer (0.3% Triton X-100, 5% fetal calf serum in PBS) for 1 h at room temperature, then incubated with primary antibodies overnight at 4°C. The primary antibodies used in this study were: Rabbit anti-Somatostatin antibody (Abclonal, A9274, 1:200), Guinea pig anti-Glucagon antibody (TAKARA, M182, 1:1,000), Mouse anti-Insulin antibody (Sino Biological, 11038-MM14-4.5, 1:1,000). Following several washes in PBST (0.3% Triton X-100 in PBS), the corresponding secondary antibodies Alexa Fluor 488-Goat anti-guinea pig (Invitrogen, A11073, 1:500), Alexa Fluor 555-Donkey anti-Rabbit (Thermofisher, A-31572, 1:500), Alexa Fluor 647-Donkey anti-Mouse (Jackson, 715605150, 1:500) and DAPI (Sigma, 28718903,1:000), diluted in blocking buffer were applied to the sections and incubated for 2 h at room temperature. The slides were then washed several times in PBST and mounted with Mowiol medium. Immunofluorescence images were acquired using a confocal microscope (ZEISS, LSM980).

### 2.4 H&E staining

Pancreatic tissue from adult axolotls were collected and fixed in MEMFA overnight at 4°C. After dehydration using a graded series of ethanol (50%, 70%, 95% and 100% diluted in PBS) and clearing in xylene, the samples were embedded in paraffin, and 5 μm sections were collected. For H&E staining, the sections were dried at 65°C for 1 h, treated with xylene to remove the paraffin, rehydrated in decreasing concentrations of ethanol, and finally in distilled water. The sections were incubated in hematoxylin solution for 5 min. After several washes with distilled water, the sections were sequentially incubated in 1% hydrochloric acid in ethanol for 10 s, washed gently with running water for 15 min, and then incubated in eosin solution for 1 min. The stained sections were dehydrated with ethanol and transferred into xylene, then mounted with neutral resin. Once the resin dried, the sections were imaged using an inverted microscope (Olympus, IX83).

### 2.5 *In situ* hybridization

To synthesize DIG-labelled or fluoresce-labelled antisense RNA probes, corresponding DNA templates containing the T7 promoter were amplified with two rounds of PCR, using axolotl pancreatic cDNA synthesized from total RNA of the pancreas as the template. The antisense RNA probes were transcribed *in vitro* using a T7 RNA polymerase transcription kit (NEB, M0251L). *In situ* hybridizations and fluorescence *in situ* hybridization were performed on 10 μm cryosections from axolotl pancreas or embryos, as previously described (Fei et al., 2017; He et al., 2020). Briefly, for *in situ* hybridizations, 500 ng/mL of DIG-labeled probes were hybridized at 60°C on 10 μm cryosections overnight. After hybridization, the cryosections were washed with a stringent buffer and incubated overnight at 4°C with anti-DIG-AP antibody (1:5,000, Roche, 11093274910). The signal was detected using BM Purple solution (Roche, 11442074001). For fluorescence *in situ* hybridization, a mixture of 300 ng/mL DIG-labeled and fluorescein-labeled RNA probes was hybridized at 60°C overnight on 10 μm cryosections. After washing with stringent buffer, the cryosections were incubated with anti-fluorescein-POD (1:500, Roche, 11426346910), and then the signal was detected using TSA fluorescein (1:50, PerkinElmer, NEL701A001KT) at room temperature. Following signal development, 3% hydrogen peroxide was used to deactivate any residual POD, and the cryosections were incubated with anti-DIG-POD (1:1,000, Roche, 11633716001) after washing. TSA Cy5 (1:50, PerkinElmer, NEL705A001KT) was used to generate a detectable signal. List of primers for RNA probe synthesis: Amy-forward: 5′-ATG​AGG​CTC​CTT​CTG​CTG​CTT​GC-3′, Amy-reverse: 5′-CTT​GTA​TTG​TTT​ATA​ACT​TG-3′, T7-Amy-reverse: 5′-TTG​AAA​TTA​ATA​CGA​CTC​ACT​ATA​GGG​CTT​GTA​TTG​TTT​ATA​ACT​TG-3′, Ins-forward: 5′-ATG​GCT​CTT​TGG​GTG​CGT​G-3′, Ins-reverse: 5′ACT​CCT​TCT​ACC​CCC​TCT​GC-3′, T7-Ins-reverse: 5′-TTG​AAA​TTA​ATA​CGA​CTC​ACT​ATA​GGG​ACT​CCT​TCT​ACC​CCC​TCT​GC-3′, Krt19-forward: 5′-ATG​TCG​TCG​AGT​ACA​GCT​TCA​GG-3′, Krt19-reverse: 5′-TTT​TCC​CCC​GCT​TTT​GGA​C-3′, T7-Krt19-reverse: 5′-ATT​ATG​CTG​AGT​GAT​ATC​CCT​CTT​TTT​CCC​CCG​CTT​TTG​GAC-3′, Ptf1a-forward: 5′-TGC​AGT​CCA​TCA​ACG​ACG​CCT​TC-3′, Ptf1a-reverse: 5′-GCG​TCA​CTG​TAA​TGA​AAG​C-3′, T7-Ptf1a-reverse: 5′-TTG​AAA​TTA​ATA​CGA​CTC​ACT​ATA​GGG​GCG​TCA​CTG​TAA​TGA​AAG​C-3′, Mln-forward: 5′-CCC​ACA​TCC​ACA​CTC​TAC​AGT​AG-3′, Mln-reverse: 5′-TCA​GTC​CAC​ATT​TTG​GTG​TTC​TG-3′, T7-Mln-reverse: 5′-TTG​AAA​TTA​ATA​CGA​CTC​ACT​ATA​GGG​TCA​GTC​CAC​ATT​TTG​GTG​TTC​TG-3′.

### 2.6 Whole-mount *in situ* hybridization

Axolotl embryos were fixed overnight in MEMFA at 4°C. Following fixation, the embryos were washed with PTW (DEPC-treated 0.1× PBS with 0.2% Tween) through a gradient of decreasing methanol concentrations (100%, 90%, 75%, 50%, and 25%). The samples were then treated with proteinase K (5 μg/mL) at room temperature for 5 min, followed by incubation at 65°C in hybridization buffer with 500 ng/mL antisense RNA probes. To remove unbound RNA probes, the samples were washed multiple times with post-hybridization buffer and MAB buffer. Next, samples were incubated with anti-DIG-AP antibody (1:5,000, Roche, 11093274910) in 1× blocking buffer (Roche, 11096176001). The signal was detected using BM Purple substrate (Roche, 11442074001). Finally, the samples were stored in 50% glycerol diluted in PBS at 4°C and imaged using an Olympus SZX10 microscope.

### 2.7 Transmission electron microscopy

TEM was conducted following standard procedures. Briefly, the isolated axolotl pancreas was cut into approximately 1 mm³ tissue blocks, which were then transferred to an EP tube containing fresh fixative for TEM (Servicebio; G1102) and fixed overnight at 4°C. After fixation, the samples were rinsed three times with 0.1M phosphate buffer (pH7.4) and post-fixed in 1.0% osmium tetroxide in phosphate buffer at room temperature for 2 h. The tissue was then dehydrated through a graded series of ethanol, embedded in Epon resin, and polymerized at 60°C overnight. Ultrathin sections were cut, mounted on copper grids, and stained with uranyl acetate and lead citrate. All grids were observed and imaged using a transmission electron microscope (Hitachi; HT7800/HT7700).

### 2.8 RNA sequencing

Total RNA was extracted from the pancreas of larvae (about 3–4 cm, 2 months old) and adult axolotls (about 25 cm, 2 years old) using TRIzol reagent (Invitrogen) and purified via LiCl precipitation. RNA sequencing was conducted on six biological samples, comprising three replicates from both the larval and adult groups. Specifically, three adults and twenty-four larvae were used for sequencing sample preparation. Three micrograms of total RNA were utilized to construct cDNA libraries according to the standard Illumina RNA-seq protocol. The generated cDNA library was sequenced using the Illumina NovaSeq6000 via a 2 × 150 bp paired-end protocol. Quality filtering and removal of residual adaptor sequences was conducted on read pairs using Trimmomatic v0.33. Salmon v1.10.0 was employed to build the axolotl genome index and guide transcript quantification. Axolotl genome assembly v6.0-DD from the axolotl-omics database (https://www.axolotl-omics.org/assemblies) (Schloissnig et al., 2021) served as the reference genome along with annotation files.

### 2.9 RNA sequencing analysis

Transcript expression levels were imported into R v4.3 and summarized at the gene level using the R/tximport v1.30.0. Gene count data were analyzed for differential expression using the Bioconductor software package DESeq2 v1.42.1. Gene expression was quantified using the transcript per million (TPM) values. Metascape was utilized for gene functional annotation. Mfuzz was applied to analyze the clustering of time series gene expression in larval and adult axolotl pancreas. For conservation analysis, RNA sequence data from untreated human samples (GSE205853) (Benaglio et al., 2022), Wt_2w and Wt_12w group of mice (GSE171251) (de Jesus et al., 2021) were compared to our adult axolotl RNA sequence data. For the identification of homologous genes, we obtained gene annotations from two sources: the human and mouse genome annotations from Ensembl, and the axolotl genome annotation (version 6.0-DD) from the axolotl-omics database. Genes that share annotations with mouse and human were considered homologous genes. Gene length and sequencing depth were standardized by using TPM values to compare homologous gene expression across the three species. |log2Fold Change| ≥ 1 with a False Discovery Rate (FDR) < 0.05 was utilized to capture meaningful changes in gene expression during development for the axolotl and mouse. To identify higher and lower expressed genes in axolotls compared to mice and humans, |log2Fold Change| ≥ 2 and FDR <0.01 were applied. Principal component analysis (PCA) was performed using the prcomp function on TPM-transformed raw counts of the most highly expressed genes across all pancreas samples. Pearson correlation coefficients were calculated to assess the correlations between the three species.

### 2.10 Glucose tolerance test and insulin tolerance test

Animals measuring about 15 cm in length were used for GTT and ITT. For the GTT experiment, axolotls were fasted for 12–16 h before being intraperitoneally injected with D-Glucose (2 mg/g) after anesthesia. Blood glucose levels were monitored at specific intervals using a glucose meter (Roche, Accu-Chek), sampling from the punctured dorsal body surface vessel. 48 h after the GTT experiment, the animals were returned to clear water for normal feeding. For the ITT experiment, axolotls were fasted for 6 h before being intraperitoneally injected with recombinant human insulin (insulin glargine; 5 μL/g) after anesthesia. Blood glucose levels were monitored at specific intervals as outlined in the GTT procedure. Similarly, following the ITT experiment, the animals were injected with 2 mg/g glucose solution and returned to clear water for normal feeding; the same concentration of glucose solution was injected for the next 2 days.

### 2.11 Statistical analysis

All axolotls were randomly assigned to different groups, and all experiments were repeated at least three times independently. For experimental data analysis, calculations were performed using GraphPad Prism 8.0.2. Unpaired, two-tailed Student’s t-tests were performed to analyze the *P* values for single comparisons between two groups, while one-way ANOVA was conducted to analyze *P* values for multiple comparisons. All data were presented as the mean ± s. e.m. Each experiment was performed blind, and no predetermination was made for sample size. *P* value < 0.05 was considered significant. *P < 0.05; **P < 0.01; ***P < 0.001; ****P < 0.0001; ns, *P* > 0.05.

## 3 Results

### 3.1 Axolotl pancreas exhibits an evolutionarily conserved structure and cell type composition

**FIGURE 1:**
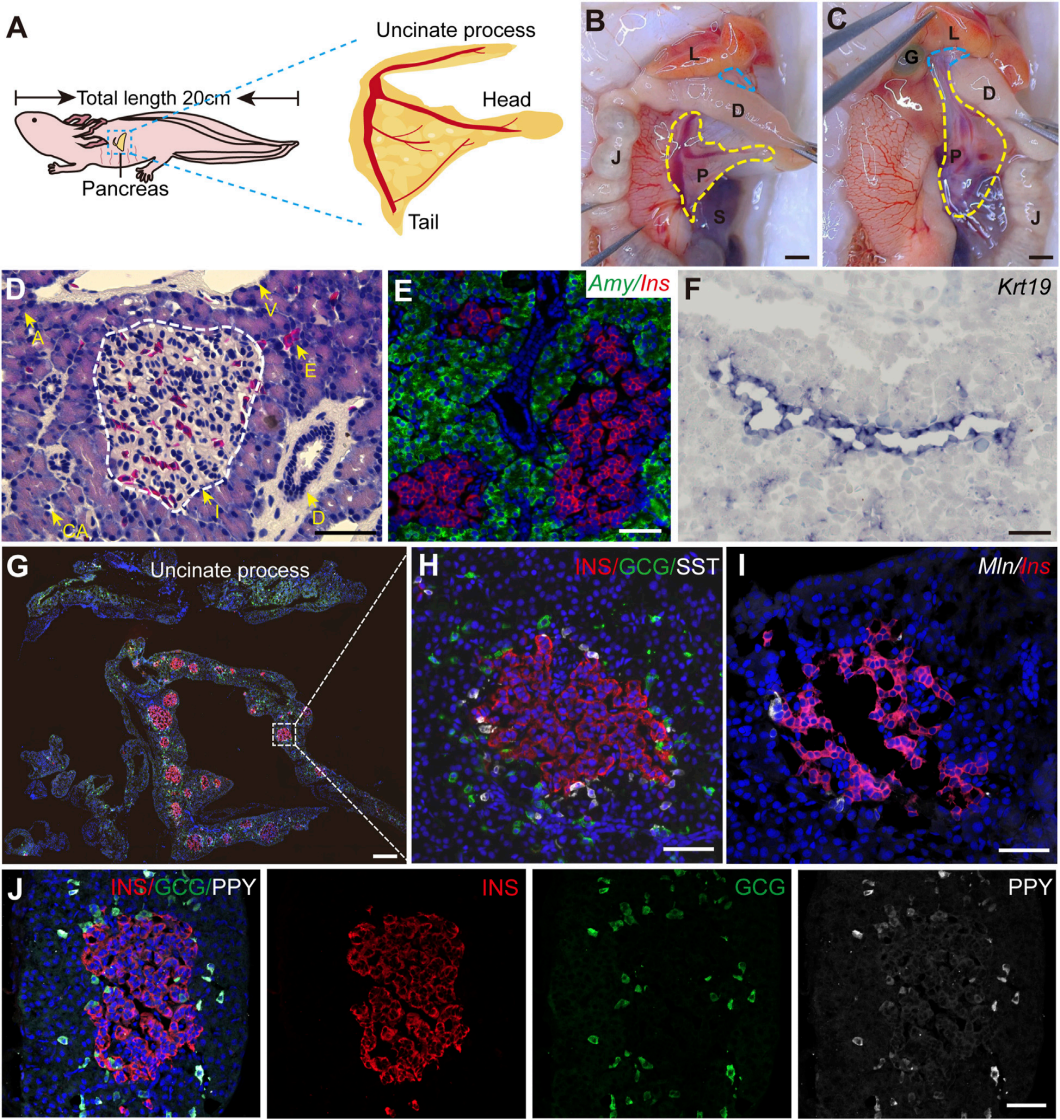
The structure of adult axolotl pancreas. **(A)** The pancreas model of an adult axolotl shows that the basic structure of the pancreas includes the head, body, tail, and uncinate process. **(B)** Anatomical structure of the ventral view of the pancreas. The pancreas head connects to the duodenum, the tail connects to the spleen, and the body is suspended from the jejunum through the mesentery. L, liver; D, duodenum; J, jejunum; P, pancreas; S, spleen; G, gallbladder. **(C)** Anatomy of the dorsal view of the pancreas. After turning the jejunum to the right, the uncinate process connects to the liver and gallbladder (blue line). **(D)** Hematoxylin-Eosin staining of the axolotl pancreas. A, acinar; I, islet; D, ductal; E, erythrocyte; CA, centroacinar. **(E–J)** *In situ* hybridization and immunofluorescence staining show the molecular features of the axolotl pancreas. **(E)** Fluorescence *in situ* hybridization for *Amylase* (*Amy*, green), and *Insulin* (*Ins*, red), combined with DAPI (blue) on pancreas paraffin sections. **(F)** *In situ* hybridization for *Keratin 19* (*Krt19*, purple) on pancreas cryosections. **(G)** Immunofluorescence for INS (red), Glucagon (GCG, green), and Somatostatin (SST, white), combined with DAPI (blue) on pancreas cryosections, showing the islets are scattered throughout the pancreas, and the uncinate process has no INS^+^ β cells. **(H)** Immunofluorescence of a magnified islet shows that the islet consists of a cluster of INS^+^ β cells (red), surrounded by GCG^+^ α cells (green) and SST^+^ δ cells (white). **(I)** Fluorescence *in situ* hybridization for *Motilin* (*Mln,* white) and *Ins* (red) on pancreas cross-cryosections shows a very small number of *Mln* ^+^ ε cells surrounding the β-cell cluster. **(J)** Immunofluorescence for Pancreatic polypeptide (PPY, white), GCG (green), and INS (red) combined with DAPI (blue) on pancreas cryosections shows PPY^+^ PP cells surround β cells and co-express with GCG^+^ α cells. Scale bar: **(B, C)** 200 μm; **(D–F, H–J)** 100 μm; **(G)** 500 μm.

To systematically characterize the axolotl pancreas, we isolated pancreas from adult axolotls and examined its morphological features, composition and distribution of pancreatic cell types. Consistent with other vertebrates, the axolotl pancreas is situated beneath the stomach. The pancreas head and tail connect to the duodenum and spleen, respectively, while the body of the pancreas is attached to the jejunum via the mesentery (Figures 1A, B). The uncinate process of the pancreas wraps around the duodenum and connects to the liver and gall bladder (Figure 1C). H&E staining of paraffin sections of the pancreas showed that islets are distributed throughout the pancreatic tissue, surrounded by exocrine tissues, including acinar cells, ducts and blood vessels (Supplementary Figure S2B; Figure 1D). *In situ* hybridization using classical pancreatic cell markers (Muraro et al., 2016) demonstrated the presence of *Ins*, a marker for endocrine β cells in islets, and *Amylase* (*Amy*) in acinar cells along with *Keratin 19* (*Krt19*) in ductal cells (Figures 1E, F) in the axolotl pancreas.

We next focused on characterizing the endocrine cells. It is well-known that pancreas contains five types of endocrine cells in mammals (Baron et al., 2016). To this end, we used specific endocrine cell markers INS, GCG, and SST to identify β, α, and δ cells respectively. Immunofluorescence on pancreatic sections showed that the main body of the pancreas (head, body, and tail) consists predominantly of INS^+^ β cells-clustered islets, surrounded by sparsely distributed GCG^+^ α cells and closely associated SST^+^ δ cells (Figures 1G, H). In contrast, the uncinate process of the pancreas exclusively contained GCG^+^ α and SST^+^ δ cells, with no INS^+^ β cells (Supplementary Figure S2A; Figure 1G). Additionally, double fluorescence *in situ* hybridization revealed that pancreatic islets composed of *Ins* ^*+*^ β cells were tightly surrounded by a limited number of *Motilin* (*Mln*)^+^ ε cells (Figure 1I). Interestingly, immunofluorescence analysis using an antibody against PPY revealed that PPY is expressed exclusively in GCG^+^ α cells (Figure 1J), suggesting the identities of α and PP cells are perhaps intermingled in axolotls. Electron microscopy showed acinar cells containing large electron-dense zymogen granules likely enriched with digestive proteins, while β cells were filled with numerous insulin granules that lacked halos, in contrast to their mammalian counterparts (Roep, 2020; Susan, 2004) (Supplementary Figures S2C, G). In addition, we also found the presence of α and δ cells in the stomach and small intestine of embryonic and juvenile axolotls (Supplementary Figure S3). In summary, axolotl pancreas, although exhibiting some unique feature, demonstrates significant structural, compositional, and subcellular similarities to other species.

### 3.2 Axolotl pancreas develops from ventral and dorsal buds

**FIGURE 2:**
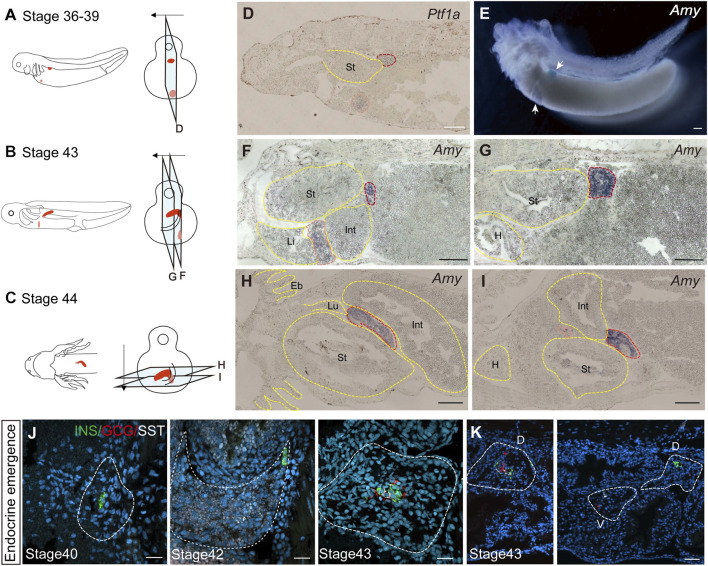
The development of the pancreas in axolotl embryos. **(A–C)** A model of pancreatic development. At stage 36, the dorsal bud and ventral bud merge. At stage 43, the dorsal bud elongates along intestinal tube. After 5 days, the dorsal bud and ventral bud fuse at stage 44. The arrows indicate the slice direction. The light blue rectangles represent sections shown in panels **(D–I)**. The red section indicates the dorsal bud, and the pink section indicates the ventral bud. **(D)** *In situ* hybridization for *Pancreas associated transcription factor 1a* (*Ptf1a,* purple) on paraffin sections of embryos shows that *Ptf1a* is expressed in both the dorsal bud (red circle) and the ventral bud (pink circle) at stage 36. **(E)** Whole-mount *in situ* hybridization shows that *Amy* is expressed in both the dorsal (blue) and ventral (purple) buds at stage 39. **(F, G)** *In situ* hybridization for *Amy* (purple) shows that the dorsal bud extends as the intestinal tube grows. **(H, I)** *In situ* hybridization for *Amy* (purple) shows that the dorsal and ventral buds have fused by stage 44. **(J)** Immunofluorescence for INS (red), GCG (green), SST (white), combined with DAPI (blue) on embryo cryosections shows that endocrine cells begin to emerge from dorsal bud. INS^+^ β cells (green) emerges at stage 40, and SST^+^ δ cells (white) and GCG^+^ α cells (red) emerging at stages 42 and 43, respectively. **(K)** Immunofluorescence shows that INS^+^ β cells are concentrated in the dorsal bud of the pancreas, while only GCG^+^ α cells (red) and SST^+^ δ cells (white) are found in the ventral bud. Eb, external branchia; St, stomach; Int, intestine; L, live; Lu, lung; H, heart. Scale bar: **(D–I)** 200 μm; **(J)** 50 μm; **(K)** 100 μm.

We next investigated the developmental origin of the adult pancreas. The differences in endocrine cell types between the uncinate process and the main portion of the axolotl pancreas suggest distinct embryonic developmental mechanism. During mammalian pancreas development, it has been reported that the ventral and dorsal pancreatic anlage give rise to the uncinate process and the main pancreas, respectively (Longnecker, 2014). To determine whether the axolotl pancreas develops from ventral and dorsal buds during the embryonic stage, we performed whole-mount and cryosection *in situ* hybridization on axolotl embryos. We detected the pancreatic ventral and dorsal buds as early as stage 36, as indicated by the expression of Pancreas associated transcription factor 1a (*Ptf1a*) and *Amy* (Figures 2A, D, E). By stage 43, the dorsal bud expanded and elongated in conjunction with the growth of the intestinal tube (Figures 2B, F, G). Eventually, the dorsal and ventral buds fused to form early pancreas a few days following stage 43 (Figures 2C, H, I). Consistent with the cell types identified in adult pancreas, immunohistochemistry analysis revealed that the β cells, δ cells and α cells in the dorsal bud started to develop at stages 40, 42 and 43, respectively (Figure 2J), while only α and δ cells were observed in the ventral bud (Figure 2K). These results indicate that the major body of the pancreas originates from the dorsal bud, whereas the uncinate process arises from the ventral bud, aligning with mammalian pancreas development.

### 3.3 The transcriptome of the axolotl pancreas exhibits evolutionary conservation and distinction

To explore the molecular characteristics of both the developing and adult pancreas, we sequenced and analyzed the gene expression profiles of total RNA extracted from 2-month-old larvae and 2-year-old adult axolotls. Our analysis identified a total of 23,931 genes expressed in the developing pancreas and 19,592 genes in the adult pancreas. Of these, 18,492 genes were commonly expressed at both stages (Supplementary Figure S4A). Gene Ontology analysis of the genes uniquely expressed in the developing pancreas (5,439 genes, with 2,634 annotated genes) revealed their primarily involvement in tissue morphogenesis and digestive system development. In contrast, the genes expressed in the adult pancreas (1,100 genes, with 565 annotated genes) were mainly associated with the positive regulation of the phosphorus metabolic process (Supplementary Figure S4B).

**FIGURE 3:**
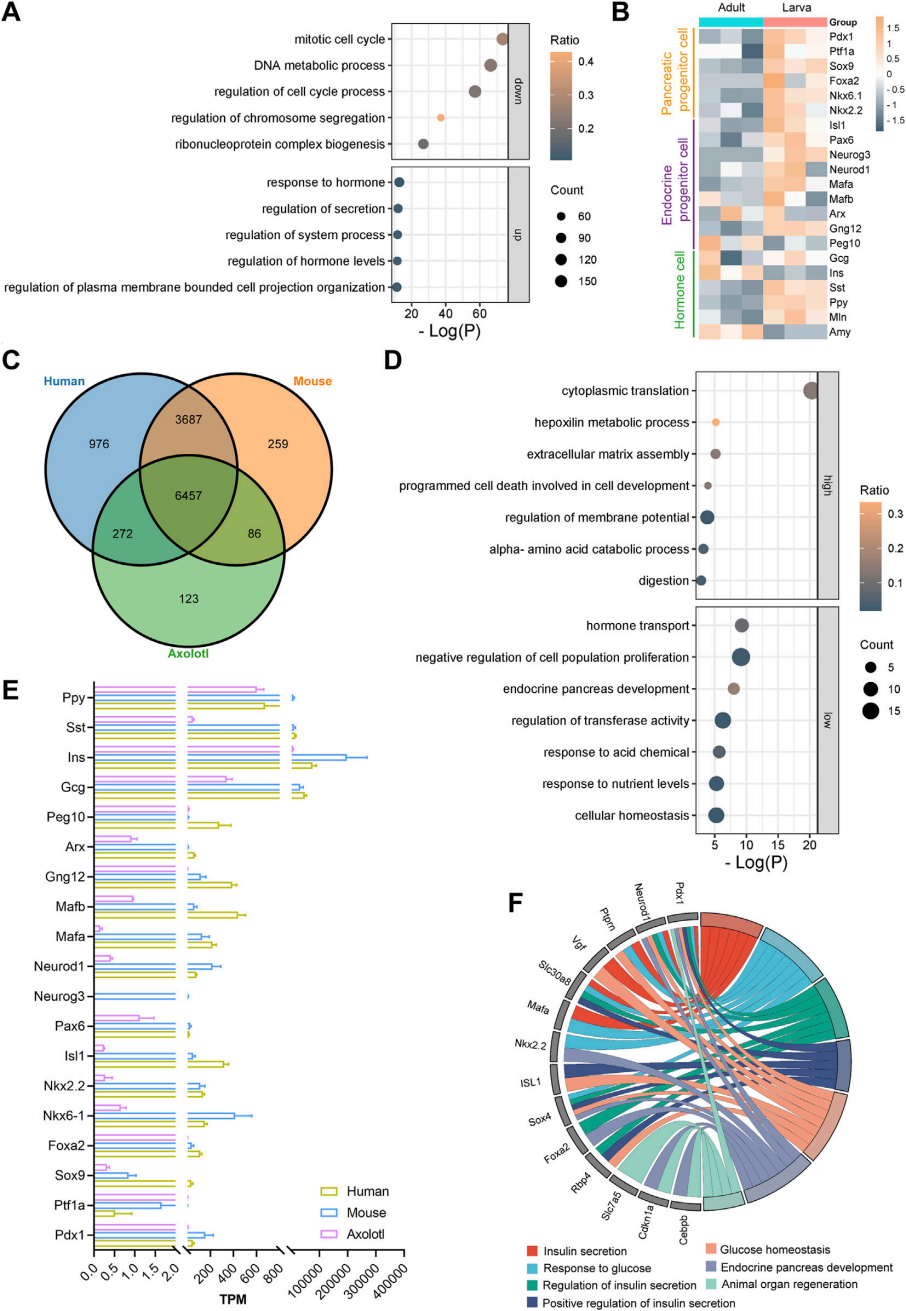
Differential gene expression analysis in the larval and adult pancreas of axolotls. **(A)** Gene Ontology (GO) analysis of the top five downregulated and upregulated genes during development shows that GO terms related to cell division, such as the regulation of mitosis, DNA metabolism, and the cell cycle, are downregulated, while GO terms related to digestion, such as response to hormone, regulation of secretion, and regulation of hormone levels, are upregulated. **(B)** Differential gene expression (DGE) analysis of the adult and larva pancreas after normalization. **(C)** Gene homology assessment of human, adult axolotls, and 12-week-old mice reveals 6,457 commonly expressed genes, which are annotated in all three species. **(D)** GO analysis of the 6,457 commonly expressed genes shows that, compared to mice and humans, axolotls have 131 genes with higher expression and 126 genes with lower expression. **(E)** Comparison of the expression levels of key pancreatic genes in adult axolotls, mice, and humans after normalization. **(F)** GO analysis of genes with low expression in axolotls reveals their involvement in processes such as insulin secretion, glucose homeostasis, endocrine development, and organ regeneration.

Further analysis on the 18,492 (16,614 annotated genes) commonly expressed genes identified 1,888 upregulated genes (1,238 annotated genes) and 2,016 downregulated genes (1,210 annotated genes) in the adult pancreas compared to the developing pancreas (Supplementary Figure S4C). Gene Ontology analysis indicated that the upregulated genes were primarily linked to hormone responses, secretion regulation, and hormone level control, while the downregulated genes were typically associated with the mitotic cell cycle, DNA metabolic processes, and cell cycle regulation (Figure 3A). Moreover, clustering of time series gene expression shows that genes involved in ribonucleoprotein complex biogenesis and RNA splicing are also highly active, in addition to those related to mitotic cell cycle phase transitions in larvae. Genes associated with signal release, peptide transport, and insulin secretion are even more active in adults (Supplementary Figure S5). These findings suggest a transition of the axolotl pancreas from an actively developing state in early stages to a homeostatic state in adulthood, indicating functional maturation during development, similar to observations in mice (de Jesus et al., 2021) (Supplementary Figures S4D, E).

The development and functional maintenance of the pancreas require coordinated gene expression regulation. We focused on well-known genes that play crucial roles during the development of pancreatic lineages (Aigha and Abdelalim, 2020; Burlison et al., 2008; Yu et al., 2021). The expression levels of markers for most pancreatic multipotent progenitor cells (*Pdx1*, *Ptf1a*, *Nkx6.1*, *Nkx2.2* and *Foxa2*), exocrine progenitor cells (*Ptf1a* and *Sox9*), and endocrine progenitor cells (*Neurog3, Isl1, Neurod1* and *Pax6*) were significantly higher in larvae compared to adult axolotls (Figure 3B). Notably, α cell fate determiners *Arx*, *MafB*, and *Peg10* exhibited comparable expression levels between larvae and adults. In contrast, β cell fate-related genes *Gng12* and *MafA* were expressed at higher levels in larvae. Functional hormone-coding genes in the pancreas, including *Ins* and *Amy*, were expressed significantly higher in adults than in larvae, while *Gcg*, *Sst*, *Ppy* and *Mln* showed lower expression levels. This regulation pattern correlates with increased demands for food digestion and metabolic homeostasis during growth. Overall, the expression patterns and dynamics of key pancreatic genes during development in juvenile and adult axolotls are conserved with those observed in mammals (Yu and Xu, 2020).

To explore the evolutionarily distinctive features of the axolotl pancreas, we compared the transcriptome of adult axolotls with those of humans and mice (Benaglio et al., 2022; de Jesus et al., 2021). Among the 19,592 genes in the adult pancreas, 11,860 were found to have homologous counterparts in both humans and mice. Among these, 6,457 genes were commonly expressed across all three species (Supplementary Figure S4F; Figure 3C). Key pancreatic genes were present in all three species, except for *Neurog3*, which is absent in both humans and axolotls (Figure 3E). We identified and analyzed differentially expressed genes exhibiting at least four-fold difference in expression levels between axolotls and mice or humans. Our results revealed that 131 genes were expressed at higher levels and 126 genes at lower levels in adult axolotls compared to the other two species (Supplementary Figure S4G). The highly expressed genes were primarily involved in cytoplasmic translation, while the lowly expressed genes were related to hormone transport and negative regulation of cell population proliferation (Figure 3D). Among the 126 downregulated genes, such as *Pdx1*, *Neurod1*, *Ptprn*, *Slc30a8*, *Rbp4*, and *Sox4*, many were associated with insulin synthesis, insulin secretion, and glucose response, indicating that axolotl pancreas may display a delayed insulin secretion response to glucose compared to mammals (Figure 3F). Therefore, we examined the expression of metabolic genes in the axolotl and found that glucose sensor genes *Slc2a1* and *Slc2a2*, insulin secretion genes *Stx1a* and *Vamp8*, and glycolytic-related kinases *Pfkl*, *Pfkm*, *Pkm*, *Gck*, *Pgk1*, and *Ldhb* are lowly expressed in the axolotl (Supplementary Figure S4H). Additionally, the genes *Slc7a5*, *Pdx1*, *Cdkn1a*, and *Cebpb* are known to be downregulated in association with positive effects on organ regeneration (Fitzner et al., 2013; Holland et al., 2005; Juran et al., 2021; Wang et al., 2022). We observed that these genes were expressed at lower levels in axolotls (Figure 3F). In conclusion, the transcriptomic characteristics of the axolotl pancreas indicate an evolutionary conservation of gene expression, suggesting that glucose regulation within pancreatic endocrine cells is less sensitive compared to mammals. Moreover, this may point to a significant regenerative potential within the axolotl pancreas.

### 3.4 Mutations of *Pdx1* cause various levels of pancreas defects but not lethality

**FIGURE 4:**
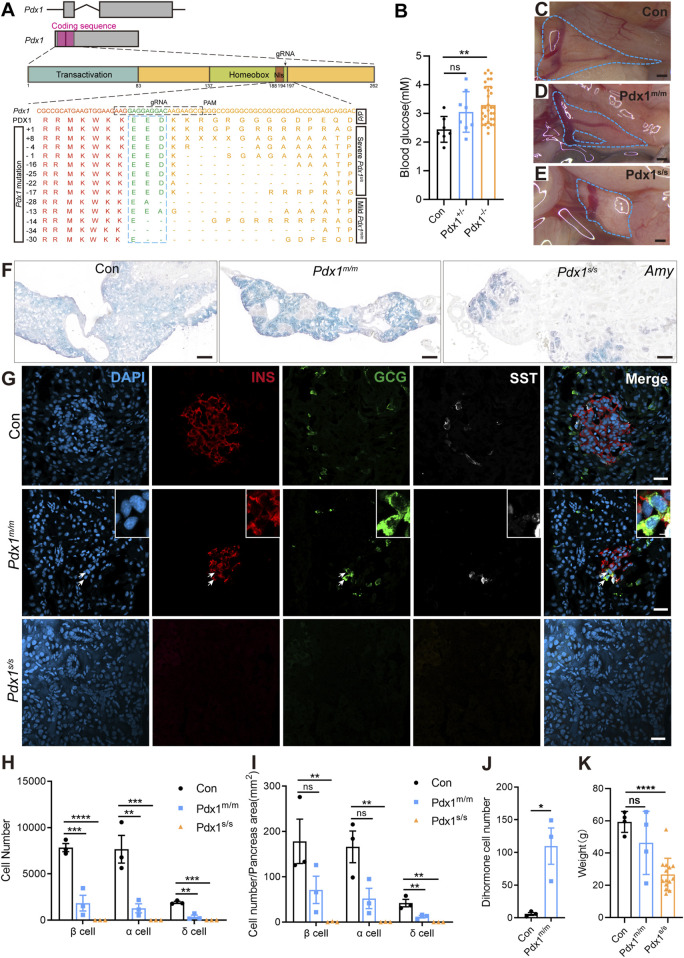
The mutations of *Pancreatic and duodenal homeobox 1* (*Pdx1*) result in varying degrees of absence in both the endocrine and exocrine cells of the axolotl pancreas, attributable to different allele mutations. **(A)** Schematic diagrams of the *Pdx1* gene structure and PDX1 protein domains, along with a schematic illustration of the actual effects of *Pdx1* mutations on the proteins. CRISPR gRNAs was designed to target the *Pdx1* sequence and injected into one-cell-stage eggs, and resulted in mild and severe phenotypes. Axolotls with a complete homeobox in both *Pdx1* alleles (*Pdx1* ^*s/s*^) exhibit a severe phenotype. In contrast, a mismatch or frameshift mutation in the homeobox region of both *Pdx1* alleles (*Pdx1* ^*m/m*^) results in a milder pancreatic defect. **(B)** Fasting blood glucose levels in control, heterozygous, and homozygous axolotls show that blood glucose significantly increase in *Pdx1* ^*−/−*^ mutants, while no significant change is observed in *Pdx1* ^*+/−*^ mutants. **(C–E)** Anatomical analysis of control **(C)** and *Pdx1* mutant axolotls (blue circle). A complete pancreatic structure, but with a small portion of the pancreas body missing **(D)**. While a severe phenotype is characterized by the development of only a remnant of pancreatic head **(E)**. **(F)** *In situ* hybridization for *Amy* (blue) on cryosections shows that *Pdx1* mutants causes a deficiency in the exocrine pancreas. **(G)** Immunofluorescence for INS (red), GCG (green), combined with DAPI (blue) on cryosections shows INS^+^/GCG^+^ double hormone cells (arrows) appear in *Pdx1* ^*m/m*^ mutant axolotls, while *Pdx1* ^*s/s*^ mutant axolotls have no endocrine cells. **(H)** Statistical analysis of endocrine cell counts shows a decrease in the number of β, α, and δ cells following *Pdx1* mutations. **(I)** Statistical analysis of the proportions of β, α, and δ cells in the control, *Pdx1* ^*m/m*^, and *Pdx1* ^*s/s*^ groups. **(J)** Statistical analysis of double hormone cells in the control and *Pdx1* ^*m/m*^ groups. **(K)** Statistical analysis shows that animal growth is severely inhibited in *Pdx1* ^*s/s*^ mutant axolotls. Data are the mean ± s. e.m. Scale bar: **(C–E)** 200 μm; **(F)** 200 μm; **(G)** 50 μm.

Evaluating gene function is crucial for understanding biological processes. Therefore, we targeted *Pdx1* to assess the functional conservation and diversity of pancreatic-specific genes. *Pdx1* is a transcription factor expressed in multipotent progenitor cells; it is essential for pancreatic development and the maintenance of β cell function (Zhu et al., 2021a; Hui and Perfetti, 2002; Stoffers et al., 1997). In mammals, *Pdx1* knockout results in severe, life-threatening pancreatic developmental defects (Fujitani et al., 2006; Jonsson et al., 1994; Schwitzgebel et al., 2003). To investigate this further, we used CRISPR/Cas9 technology to generate *Pdx1* mutants. The gRNA was designed to target the region immediately following the nuclear localization signal within the homeobox domain (Figure 4A). F_0_ founders were bred with *d/d* axolotls to produce F_1_ heterozygous embryos, in which we identified various insertions or deletions (indels) at the targeted site in germline-transmitted F_1_ animals (Figure 4A). Additionally, we performed intercrosses of individual F_0_ founders to obtain “homozygous” mutants, considering both alleles with frameshift mutations as “homozygous”.

The “homozygous” mutants survived but predominantly exhibited stunted growth and dwarfism (Figure 4K). Following a 24-h fasting period, we assessed the blood glucose levels of axolotls measuring 7 cm in length, subsequently conducting genotyping. The results indicated that the *Pdx1* double allelic mutants, as opposed to heterozygous individuals, exhibited hyperglycaemia compared to the *d/d* controls (Figure 4B). We then isolated the pancreas from 8-month-old “homozygous” mutants and sibling controls to investigate pancreatic developmental phenotypes. Based on pancreatic morphology, we classified the bi-allelic “homozygous” mutants into two categories: mild (*Pdx1* ^*m/m*^) and severe (*Pdx1* ^*s/s*^) phenotypes. The pancreas of *Pdx1* ^*m/m*^ mutants showed relatively normal in size and morphology, while that of *Pdx1* ^*s/s*^ mutants was significantly reduced, consisting only of a dense remnant of the pancreatic head (Figures 4C–E).

mRNA *in situ* hybridization using an antisense *Amy* probe revealed a notable reduction in exocrine tissue in “homozygous” mutants, particularly pronounced in *Pdx1* ^*s/s*^ mutants, alongside an increased proportion of ductal structures (Figure 4F). Further immunofluorescence analysis using antibodies against INS, GCG and SST demonstrated a decrease in endocrine cells in *Pdx1* ^*m/m*^ mutants and a near-total depletion in *Pdx1* ^*s/s*^ mutants (Figures 4G–I). Intriguingly, the presence of INS^+^/GCG^+^ dihormonal cells and a small number of GCG^+^/SST^+^ dihormonal cells, which were nearly absent in 8-month-old *d/d* individuals, was noted in *Pdx1* ^*m/m*^ mutants (Figure 4J; Supplementary Figure S6). These findings underscore the critical role of *Pdx1* in pancreas development and blood glucose regulation in axolotls, consistent with observations in mammals (Offield et al., 1996; Oliver-Krasinski et al., 2009). In summary, *Pdx1* mutant axolotls exhibit pancreatic developmental defects and a reduction in both endocrine and exocrine cell populations, characteristics similar to those observed in mammals. However, unlike mammals (Jonsson et al., 1994), these mutant axolotls can survive for long period, despite exhibiting severe pancreatic defects.

### 3.5 GTT and ITT reveal a slow blood glucose metabolism and a delayed insulin response in axolotl pancreas

**FIGURE 5:**
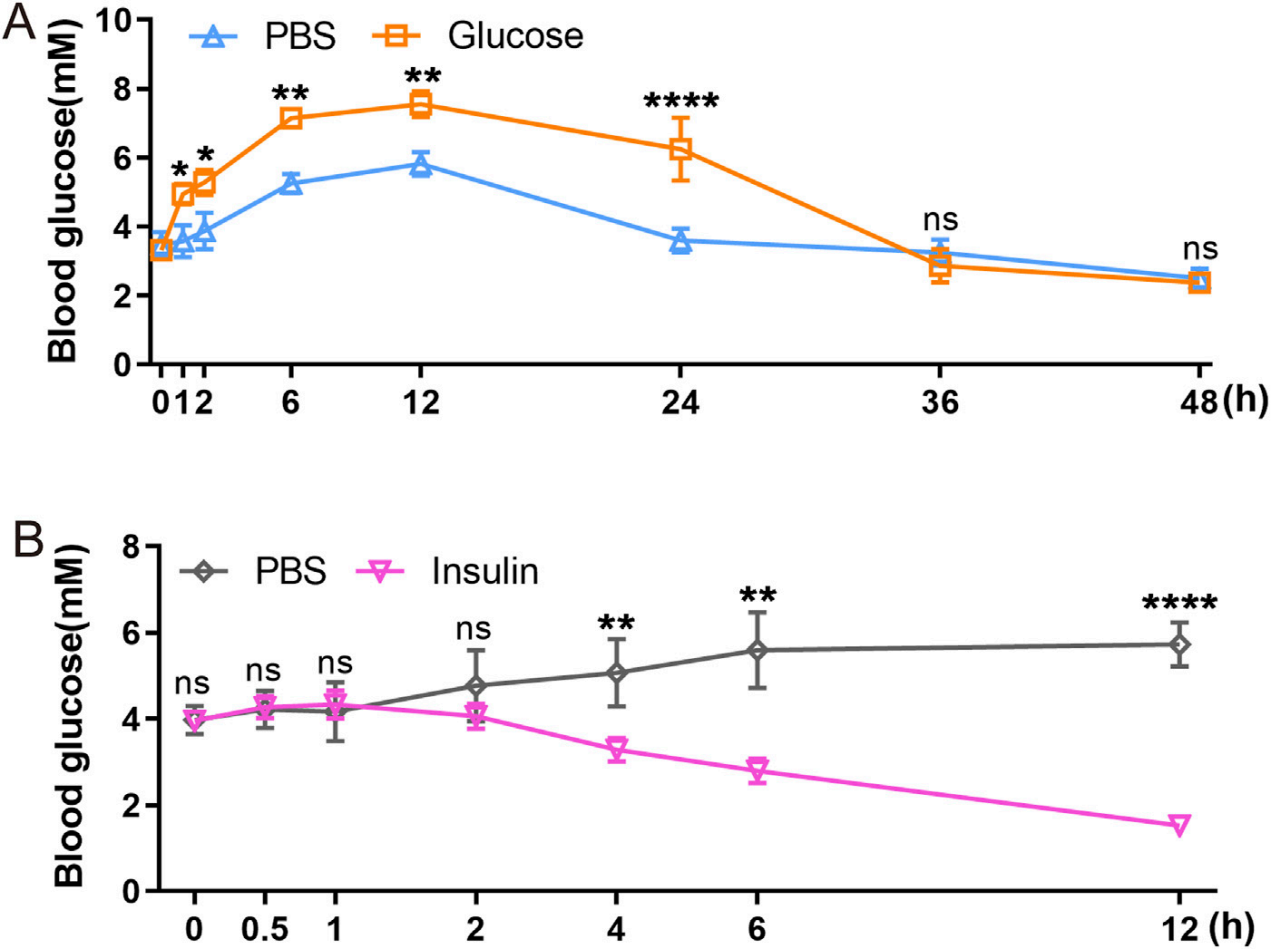
Functional characterization of axolotl islets. **(A)** The glucose tolerance test shows that blood glucose levels gradually rise between 0 and 12 h post-injection of a 2 mg/g glucose, followed by a subsequent decrease. **(B)** The insulin tolerance test shows that blood glucose levels gradually decrease after injection of a 5 μL/g insulin. Data are the mean ± s. e.m.

GTT and ITT approach are standard methods for assessing glucose metabolism and insulin response. We employed intraperitoneal GTT and ITT to characterize these processes in axolotls. Assessing these processes in smaller models like zebrafish is challenging, so it is particularly relevant to determine their feasibility in axolotls. We initially followed established procedures used in mice (Cassano et al., 2020; Qu et al., 2019; Zhu et al., 2021b), but noted unexpected fluctuations in glucose and insulin levels during the measurement periods. Considering the evidence of the slower glucose metabolism in axolotls suggested by sequencing data, we optimized our experimental protocols for intraperitoneal GTT and ITT. Blood glucose levels were measured at various time points: 0, 1, 2, 6, 12, 24, 36 and 48 h following a 2 mg/g glucose injection during GTT (Figure 5A). In comparison to blood glucose measurement sampling in mice (Small et al., 2022), the intervals for measurements in axolotls are longer. Our findings revealed that the average fasting blood glucose level in adult axolotls was 3.317 ± 0.419 mmol/L 12 h after an intraperitoneal injection of glucose (2 mg/g), blood glucose levels rose to 7.55 ± 0.369 mmol/L. By 36 h post-injection, glucose levels returned to basal level (2.867 ± 0.486 mmol/L). ITT was performed on 6 h fasted axolotls. 5 μL/g insulin glargine was injected into the axolotl, and blood glucose levels were measured at 0, 0.5, 1, 2, 4, 6 and 12 h after insulin injection (Figure 5B). The results showed that blood glucose levels decreased from 3.962 ± 0.134 mmol/L at baseline to 1.525 ± 0.207 mmol/L at the 12-h mark, where levels stabilized. In summary, axolotls exhibit lower blood glucose homeostasis compared to mammals, aligning with the notion that blood glucose levels correlate positively with metabolic rates (Simon et al., 2019; DeFronzo et al., 1979). This aligns with gene expression analysis and further indicates slower glucose metabolism in axolotls.

## 4 Discussion

In this study, we investigated the fundamental cellular and molecular features of the axolotl pancreas during development and homeostasis. Our findings reveal that axolotl pancreas exhibits typical evolutionary transitional characteristics, positioning it between the rodents and teleost. Notably, the axolotl pancreas also possesses unique traits, such as a slow glucose metabolic rate and distinct endocrine cell state. Furthermore, the ability to conduct GTT and ITT in individual axolotls, combined with their regenerative potential, makes this species a promising model for pancreas research.

During pancreatic evolution, *Protochordates* such as amphioxus lack a pancreas but possess enteroendocrine cells, including Ins^+^ and Sst^+^ cells (Dai et al., 2024). There are GCG^+^ and SST^+^ cells present in axolotl’s stomach and intestines, but no INS^+^ cells (Supplementary Figure S3). Basally-branching vertebrates such as lampreys exhibit a single-hormone (Ins^+^) islet organ during larval stages, which later develops into a three-hormone (Ins^+^, Sst^+^ and Ppy^+^) islet organ in adults (Youson and Al-Mahrouki, 1999). In *Osteichthyes* (e.g., lungfish, zebrafish), the pancreas is located near the anterior and dorsal part of the intestine, with the islets encapsulated by exocrine cells (Icardo et al., 2010; Joss, 2010). The zebrafish is a commonly used model organism widely employed in genetic and embryonic development research (Veldman and Lin, 2008). As a tetrapod amphibian, the evolution of the axolotl pancreas occupies an intermediate position between zebrafish and mammals (Boisvert et al., 2013). Unlike zebrafish, where the ventral bud solely contributes to the exocrine pancreas, both buds in axolotls can differentiate into endocrine and exocrine tissues, similar to mammals (Pan and Wright, 2011). The expression of *Ptf1a*, a pivotal transcription factor for exocrine formation, is localized in the ventral bud of zebrafish (Matsuda, 2018; Lin et al., 2004). However, axolotls retained the ability to express *Ptf1a* in both buds. It appears that the dorsal bud of the pancreas gradually acquired the capability to develop into both endocrine and exocrine glands during evolution. Although both buds contribute to pancreatic endocrine lineages, the ventral bud in the axolotl does not contribute to β cell production, indicating its transitional position between the fish and mice. Additionally, axolotls have large islets similar to those in mammals, unlike the β cells of *Tilapia*, which are found in Brockmann bodies or isolated islets along the mesentery, lacking surrounding exocrine tissue (Moss et al., 2009; Xu et al., 2004).

Certain pancreatic cells in axolotls exhibit distinct characteristics that differentiate them from their mammalian counterparts. For instance, PPY and GCG are co-expressed in axolotl pancreas. This phenomenon has been reported in various simple vertebrates, such as *Dipnoiformes* and *Lepisosteus osseus* (Groff and Youson, 1998). During pancreatic development in mice, PP cells and α cells share a common progenitor, indicating that PP cells and α cells differentiate into two distinct cell types during evolution. In previous studies, PPY^+^/GCG^+^ bihormonal cells have been reported (Huang et al., 2009; Aragón et al., 2015). In adult wild-type mouse islets, approximately 18.2% of PPY^+^ cells are PPY^+^/GCG^+^ bihormonal cells. In contrast, in human non-diabetic islets, only 0.1%–0.5% of PPY^+^ cells are PPY^+^/GCG^+^ bihormonal cells, a proportion lower than in mice. This suggests that as species evolve, the differentiation of the endocrine lineage becomes more refined to achieve precise regulation. The co-expression of PPY and GCG in the axolotl pancreas suggests the presence of a unique regulatory mechanism that may influence endocrine cell function and glucose homeostasis. It is well known that PPY regulates pancreatic secretion through both endocrine and exocrine tissues (Kojima et al., 2007), while GCG mainly regulates glucose metabolism (Unger, 1971). Their co-expression in the same cell may indicate a coordinated regulation of metabolic and digestive functions, particularly in response to metabolic demands or tissue repair during development or regeneration. Perez-Frances and colleagues reported that bihormonal cells are upregulated after injury and can contribute to endocrine cell populations (Perez-Frances et al., 2021). Therefore, the presence of PPY^+^/GCG^+^ bihormonal cells may serve as a reservoir of pancreatic islet stem cells, and when endocrine function is compromised, these bihormonal cells may act as progenitor cells during development, promoting the regeneration of other islet lineages.

Additionally, ε cells in axolotls do not express the marker GHRL but instead contain a GHRL-like peptide, MLN, believed to have evolved from the same ancestral gene as GHRL (Asakawa et al., 2001; Hara et al., 2018). In zebrafish, Ghrl and Mln are co-expressed in ε cells, whereas Mln is absent in rats and mice (Kawamura et al., 2019; Lavergne et al., 2020). Given the roles of both peptides in regulating gastrointestinal motility and appetite--critical for nutrient digestion and absorption (Kitazawa and Kaiya, 2019; Peeters, 2005), we designated the MLN^+^ pancreatic cells as ε cells in axolotls. Unlike the direct contact observed between GCG^+^ α cells and SST^+^ δ cells with β cells in humans (Abdulreda et al., 2013), most surrounding endocrine cells in axolotls maintain a noticeable distance from β cell clusters. Although the precise implications of this spatial arrangement are unknown, it suggests that endocrine cell functions in axolotls may not be as tightly regulated as in mammals.

*Pdx1* is expressed in multipotent progenitor cells of the pancreas and is crucial for maintaining the identity and function of β cells by repressing the reprogramming of α cells in adulthood (Gao et al., 2014). In mice, a homozygous deletion of the second exon of *Pdx1* results in the failure of pancreatic development and death within a few days after birth due to hyperglycemia (Offield et al., 1996; Jonsson et al., 1994). In axolotls, following *Pdx1* mutation, the pancreatic area in F_0_ chimeras was reduced, but the number of endocrine cells remained unaffected, and only a few individuals exhibited an increase in blood glucose. In contrast, F_1_ “homozygous” mutants managed to survive despite a nearly absent pancreatic tissue structure, although they were reduced in size compared to controls, reminiscent of pancreas agenesis, neonatal diabetes, and delayed fetal development resulting from *Pdx1* homozygous loss in humans (Schwitzgebel et al., 2003). The *Pdx* family consists of two members, *Pdx1* and *Pdx2*, with reports suggesting that *Pdx2* has been lost in tetrapod (Mulley and Holland, 2010). Our sequencing data and the previously assembled axolotl genome support the absence of *Pdx2* in axolotls (Schloissnig et al., 2021; Smith et al., 2019). Thus, the loss of *Pdx1* is unlikely to be compensated by a homologous gene, implying that other compensatory mechanisms may exist. Given that the knockout target site in *Pdx1* is at the end of the DNA-binding domain, following the nuclear localization signal, and considering *Pdx1*’s many binding sites that regulate pancreatic development, we hypothesize that a complete DNA-binding domain with a 3′UTR frameshift may permit PDX1 to enter the nucleus and function in a “dominant-negative” manner, resulting in a more severe phenotype. In contrast, deletion at the end of the DNA-binding domain could inhibit PDX1 from entering the nucleus, allowing other compensatory transcription factors to function, resulting in a milder phenotype. However, these transcription factors may not regulate endocrine differentiation as precisely as PDX1, potentially leading to an increase in dihormonal cells.

Metabolism in axolotls is characterized by sluggishness, as evidenced by the attenuated expression levels of glucose sensor genes, insulin secretion genes, and glycolytic-related kinases. In addition, the expression of K^+^/Ca^2+^ ion channel protein genes *Cbarp*, *Abcc8* and *Kcnj11*, and genes involved in glucose metabolism signaling pathways, such as *Akt1/2*, *Prkaa1*, and *Stk11*, are also downregulated in axolotls (Supplementary Figure S4H). This diminished expression renders axolotls less sensitive to glucose and insulin, leading to sluggish blood glucose regulation, as shown by our ITT and GTT results. Some studies suggest that food intake or glucose infusion can promote β cell replication and pluripotent stem cells differentiation (Alonso et al., 2007; Bonner-Weir et al., 1989; Wang et al., 2024), while insulin-resistant states lead to a compensatory increase in β cell mass (Kulkarni et al., 2004). We hypothesize that the unique characteristics of glucose metabolism observed in axolotls may enhance β cell regeneration, warranting further investigation into the mechanisms underlying β cell regeneration.

In summary, we discuss both common and unique pancreatic features between axolotl and other species. The axolotl serves as a valuable model for enriching our understanding of the relationship between ontogeny and phylogeny. Furthermore, the axolotl is well-known for its exceptional regenerative abilities, particularly in limbs, brains and spinal cord. Therefore, studying the regeneration of the axolotl pancreas is highly meaningful. Given the axolotl’s regenerative capacity, future research could investigate its ability to regenerate the pancreas following injury. Exploring stem cell or progenitor sources, the signaling pathways, and transcriptional networks involved in pancreatic regeneration in axolotls could provide valuable insights into tissue repair mechanisms and offer new avenues for regenerative therapies, especially for conditions like diabetes. This represents an exciting direction in the field of regenerative biology, building on the foundation laid by this work.

## Data Availability

The datasets presented in this study can be found in online repositories. The name of the repository is the China National Center for Bioinformation’s Genome Sequence Archive (GSA), and the accession number is CRA019924: https://ngdc.cncb.ac.cn/gsa/s/519FVvW5.

## Glossary

Nkx6.1: NK6 Homeobox 1
Nkx2.2: NK2 Homeobox 2
Foxa2: Forkhead Box A2
Sox9: SRY-Box Transcription Factor 9
Neurog3: Neurogenin 3
Isl1: ISL LIM Homeobox 1
Neurod1: Neuronal Differentiation 1
Pax6: Paired Box 6
Arx: Aristaless Related Homeobox
MafB: MAF BZIP Transcription Factor B
Peg10: Paternally Expressed 10
Gng12: G Protein Subunit Gamma 12
MafA: MAF BZIP Transcription Factor A
Ptprn: Protein Tyrosine Phosphatase Receptor Type N
Slc30a8: Solute Carrier Family 30 Member 8
Rbp4: Retinol Binding Protein 4
Sox4: SRY-Box Transcription Factor 4
Slc2a1: Solute Carrier Family 2 Member 1
Slc2a2: Solute Carrier Family 2 Member 2
Stx1a: Syntaxin 1A
Vamp8: Vesicle Associated Membrane Protein 8
Pfkl: Phosphofructokinase, Liver Type
Pfkm: Phosphofructokinase, Muscle
Pkm: Pyruvate Kinase M1/2
Gck: Glucokinase
Pgk1: Phosphoglycerate Kinase 1
Ldhb: Lactate Dehydrogenase B
Slc7a5: Solute Carrier Family 7 Member 5
Cdkn1a: Cyclin Dependent Kinase Inhibitor 1A
Cebpb: CCAAT Enhancer Binding Protein Beta
Cbarp: CACN Subunit Beta Associated Regulatory Protein
Abcc8: ATP Binding Cassette Subfamily C Member 8
Kcnj11: Potassium Inwardly Rectifying Channel Subfamily J Member 11
Akt1/2: AKT Serine/Threonine Kinase 1/2
Prkaa1: Protein Kinase AMP-Activated Catalytic Subunit Alpha 1
Stk11: Serine/Threonine Kinase 11
