# Supplementary material for: Revealing the biological features of the axolotl pancreas as a new research model
Source: Front Cell Dev Biol. 2025 Jan 31;13:1531903. doi: 10.3389/fcell.2025.1531903 (PMC11825805; doi:10.3389/fcell.2025.1531903)
Supplement: Supplementary file 1 [file DataSheet1.pdf]

Supplementary Material

Supplementary Figures

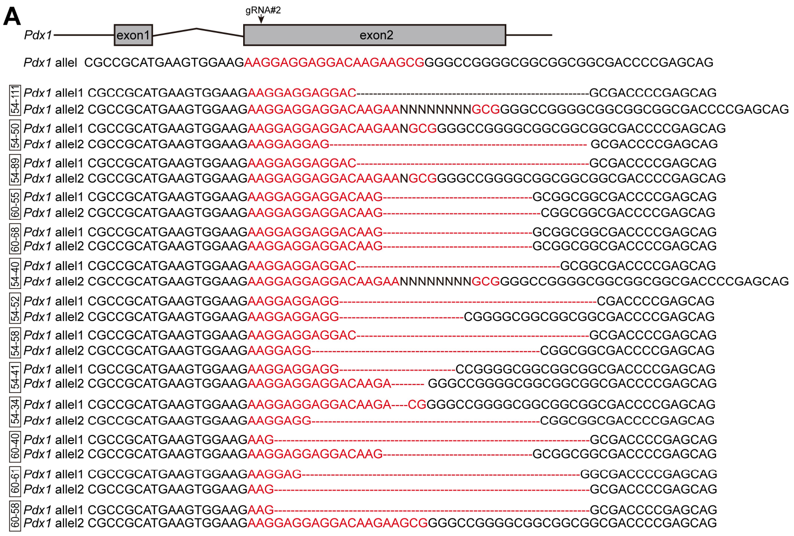

B

F0 animal	Indel (%)
Pdx1-exon2-gRNA#1	95
Pdx1-exon2-gRNA#2	81
Pdx1-exon2-gRNA#3	81
Pdx1-exon2-gRNA#4	92

C

F1 animal ID	Indel (%)	Genotype	Parental information
54-111	83	-25/+8	54- are from *Pdx1*-exon2-gRNA#2 crossed with *Pdx1*-exon2-gRNA#3. 60- are from *Pdx1*-exon2-gRNA#1 crossed with *Pdx1*-exon2-gRNA#4.
54-50	91	-28/+1	
54-89	96	-25	
60-55	97	-16/-17	
60-68	100	-16	
54-40	86	-22/+8	
54-52	94	-28/-14	
54-58	93	-28/-25	
54-41	94	-13/-4	
54-34	86	-25/-2	
60-40	86	-34/-16	
60-61	89	-34/-30	
60-58	57	-34/0	

Supplementary Figure S1. The genotype of the *Pdx1* mutant axolotls used in the experiment. **(A)** Schematic diagrams of the *Pdx1* gene structure and the genotypes of the experimental axolotls. **(B)** Different types of indel mutations in F_0 axolotls. **(C)** Different types of indel mutations in F_1 axolotls.

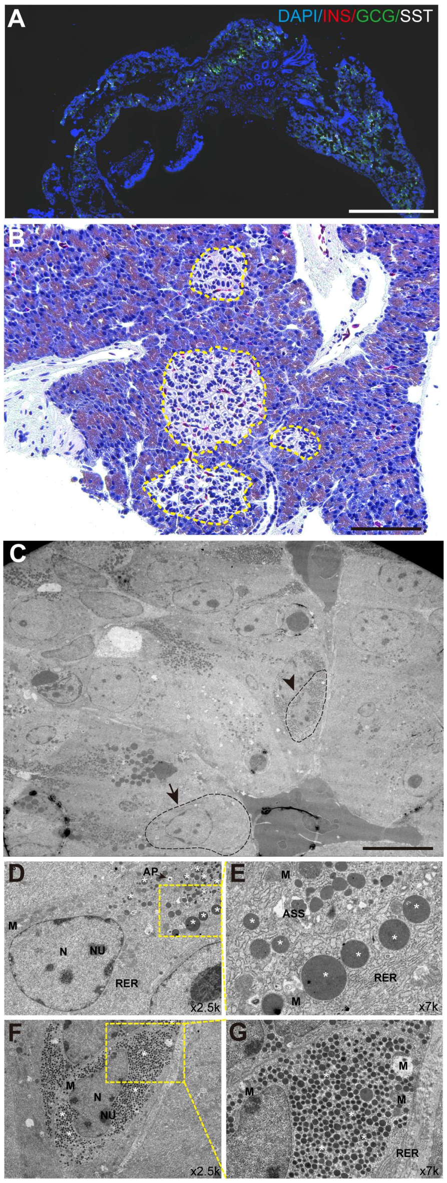

Supplementary Figure S2. The structure of adult axolotl pancreas. (A) Immunofluorescence for INS (red), GCG (green), and SST (white), combined with DAPI (blue) on pancreas cryosections. Note the uncinate process has no INS^{+} β cells. Scale bar: 1 mm. **(B)** Hematoxylin-Eosin staining of the axolotl pancreas. Note the islets are scattered throughout the pancreas. Scale bar: 200 µm. (C-G) Electron microscopy of adult pancreas sections shows the distinct cellular structures. **(C)** The arrow indicates an acinar cell and the arrowhead indicates a β cell. Scale bar: 20 µm. **(D-E)** Magnified views of an acinar cell shows large zymogen granules in the cell (arrow in panel C). **(F-G)** Magnified views of β cell shows abundance of insulin vesicles (arrowhead in panel D). Key structures include:Nucleus (N); Nucleolus (NU); Mitochondria (M); Rough endoplasmic reticulum (RER); Zymogen granules (ZG, indicated with an asterisk); Autophagosome (AP); Autophagolysosome (ASS).

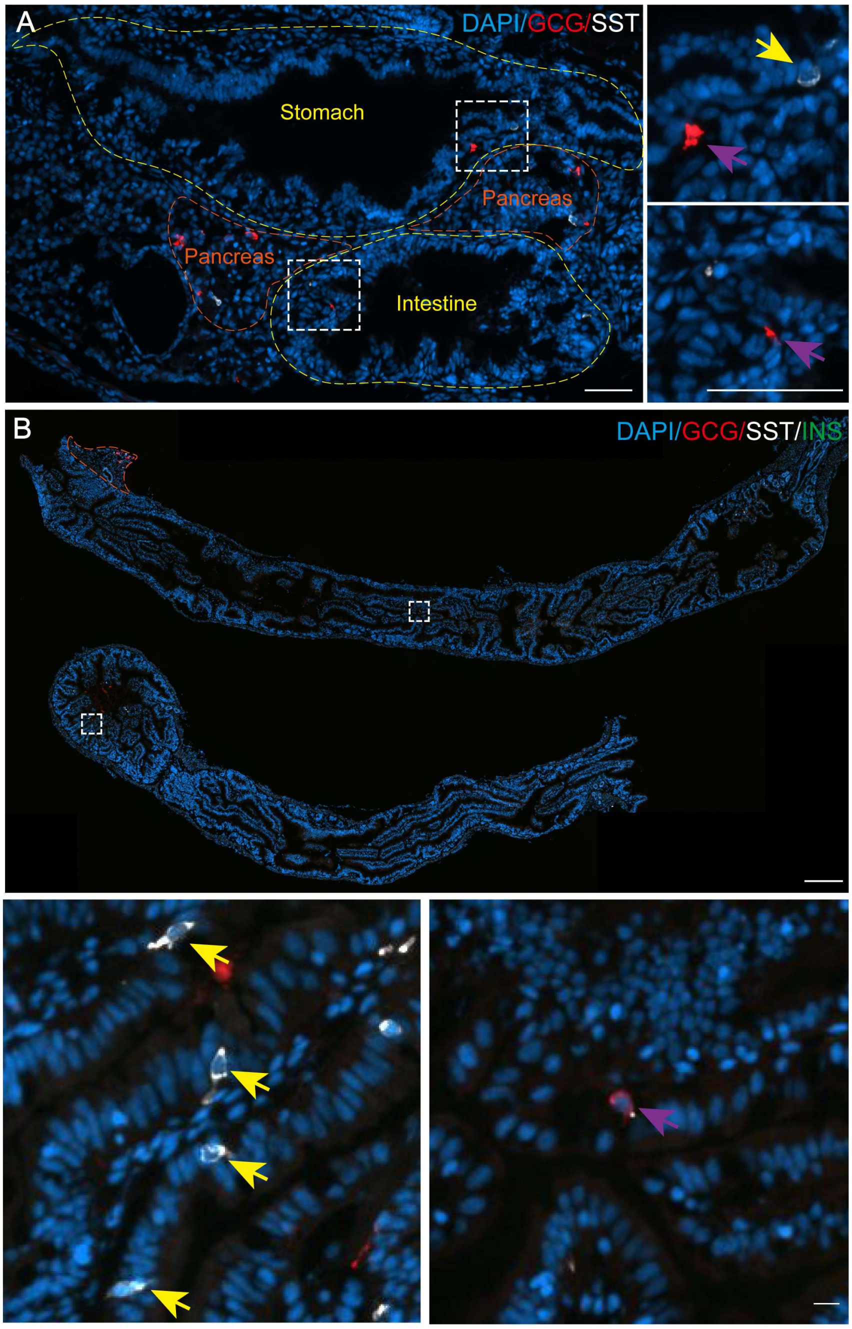

Supplementary Figure S3. GCG^{+} and SST^{+} cells present in axolotl's stomach and intestine. (A) Immunofluorescence for GCG (red) and SST (white) combined with DAPI (blue) on cryosections in stage44 embryo shows GCG^{+} cells (purple arrows) and SST^{+} cells (yellow arrows) present in the stomach. Scale bar: 100 µm. **(B)** Immunofluorescence for INS (green), GCG (red), and SST (white) combined with DAPI (blue) on cryosections in 6 cm axolotl's intestine shows GCG^{+} and SST^{+} cells present in the intestine. Red dashed lines indicate the pancreas areas; yellow dashed lines indicate the stomach and intestine. Scale bar: 500 µm; Scale bar of magnified views: 20 µm.

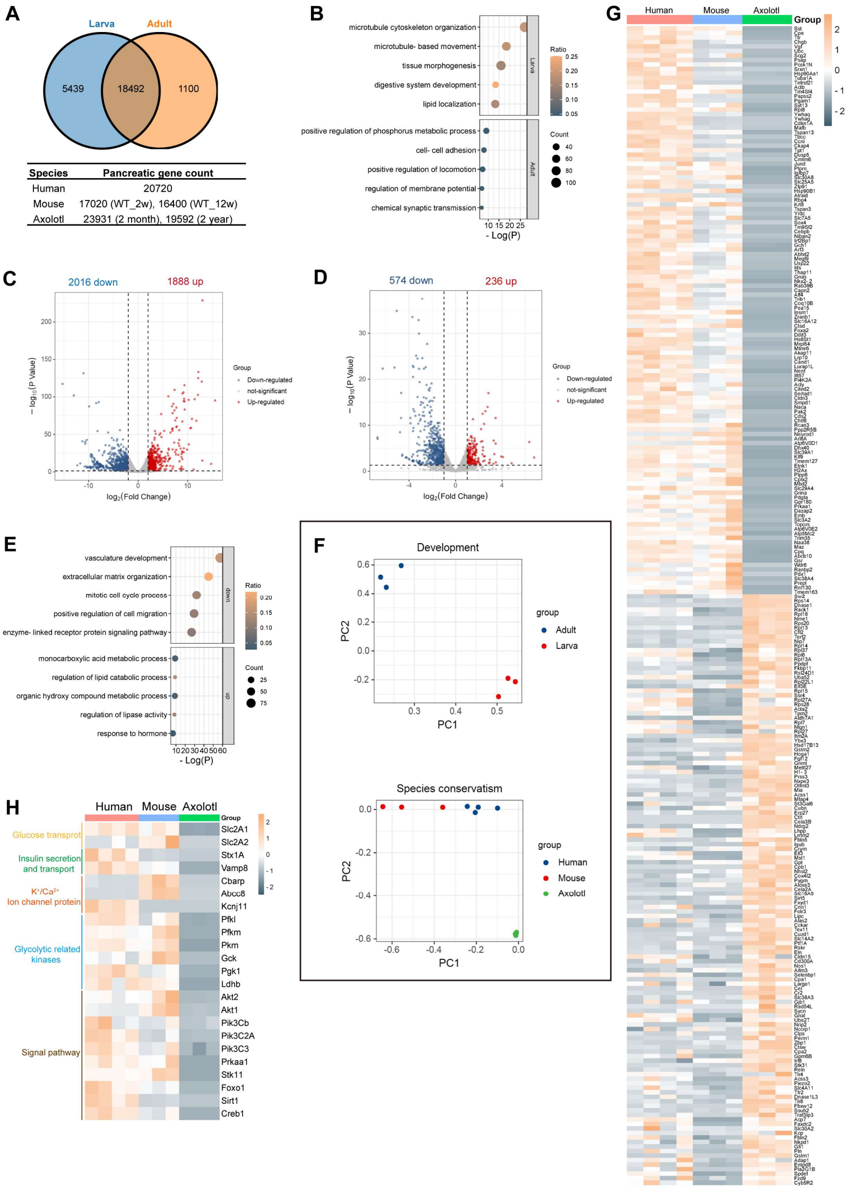

Supplementary Figure S4. Differential RNA-seq analysis across developmental stages and species reveals gene expression changes and biological process enrichment. (A) RNA-seq in adults (2 years) and larvae (2 months) shows that 18,492 genes are expressed during development, with 5,439 genes silenced and 1,100 genes activated. **(B)** The top five enriched biological processes for genes specifically expressed in adults and larvae. **(C)** DGE analysis reveals that 1,888 genes are upregulated and 2,016 genes are downregulated in adult axolotls compared to the larval stage. **(D)** DGE analysis in mice shows 236 genes are upregulated and 574 genes are downregulated in adults compared to the babies. **(E)** Biological processes are significantly enriched for genes specifically expressed in 2-week-old mice compared to those in 12-week-old mice, showing increased metabolic activity and decreased cell division in the pancreas during development. **(F)** Principal component analysis (PCA) across three species reveals the differences and clustering patterns among humans, mice, and axolotls. **(G)** Differential RNA analysis of genes with higher and lower expression in axolotls compared to humans and mice. **(H)** Differential RNA analysis of genes related to glucose metabolism and insulin secretion was conducted in humans, mice, and axolotls.

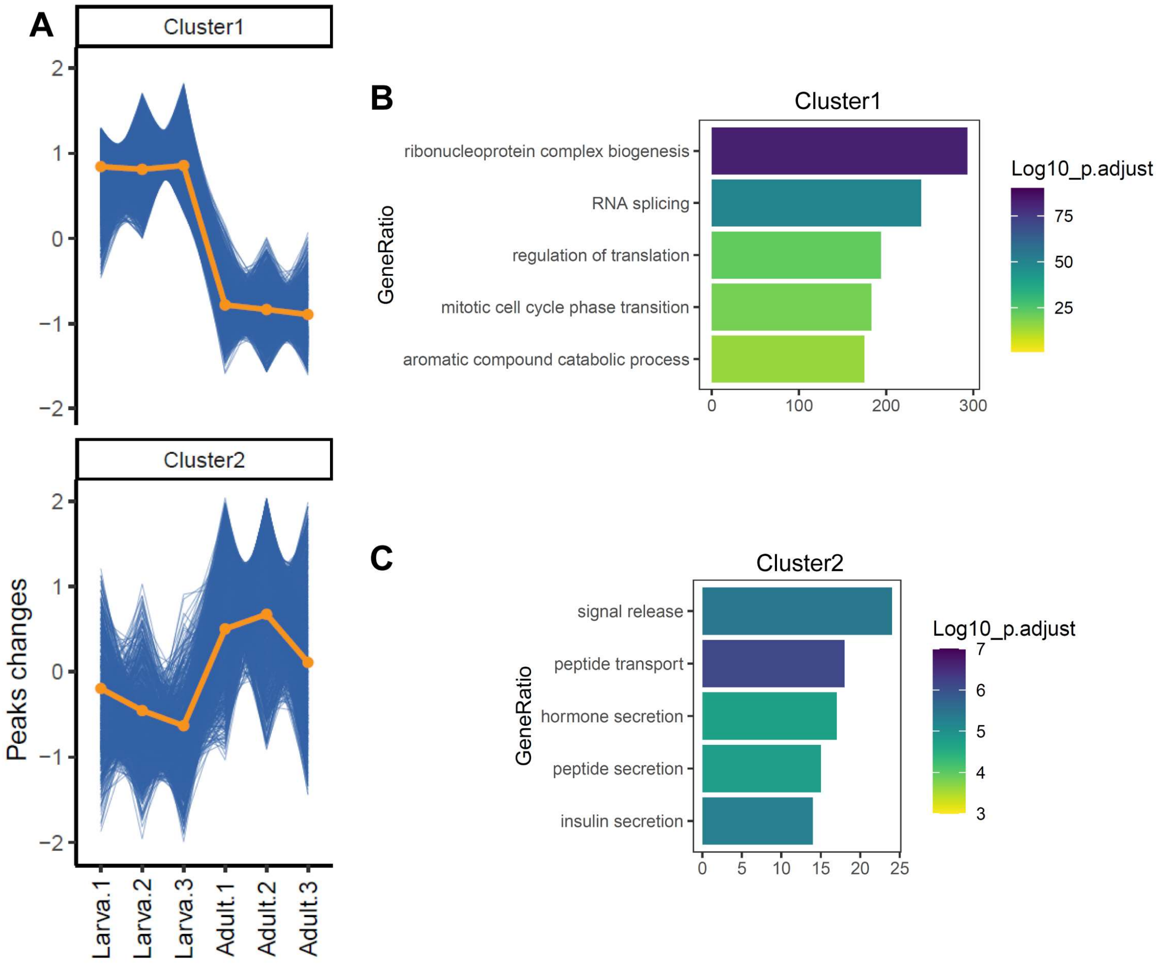

Supplementary Figure S5. Time series gene expression (Mfuzz) for identifying clusters of genes whose expression changes between the larval and adult stages. (A) Cluster 1 and Cluster 2 represent the gene sets that are upregulate in larva and adult axolotls, respectively. **(B)** GO analysis of genes upregulate in Cluster 1. **(C)** GO analysis of genes upregulate in Cluster 2.

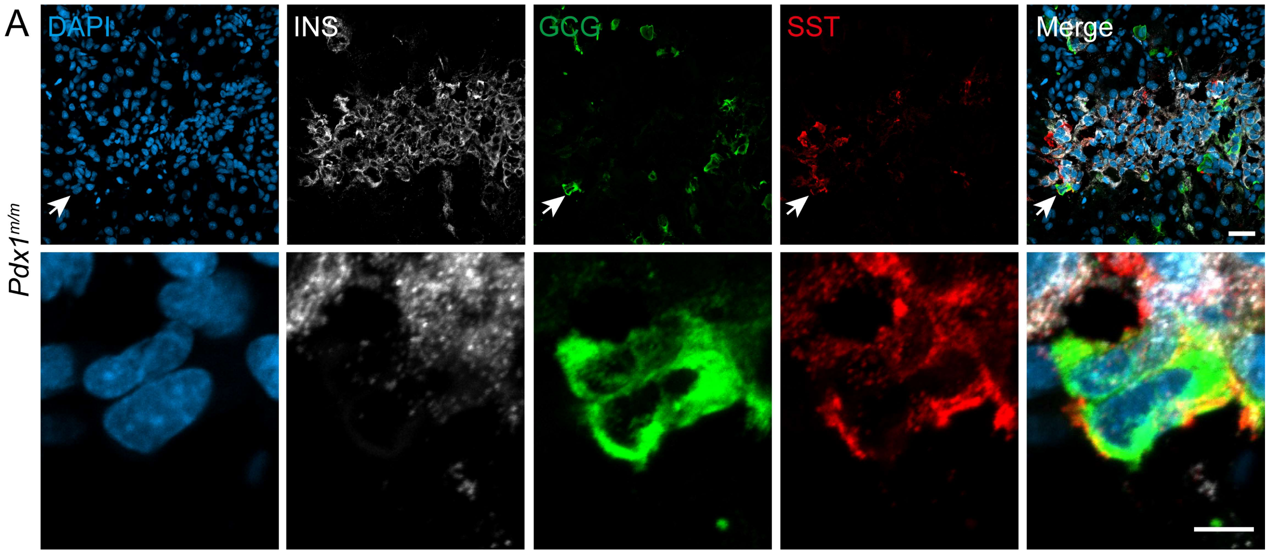

Supplementary Figure S6. Dihormonal cells appear in *Pdx1$^{m/m}$* mutant axolotls. (A) Immunofluorescence for INS (white), GCG (green), and SST (red) combined with DAPI (blue) on pancreas cryosections in *Pdx1$^{m/m}$* axolotls shows GCG^{+} / SST^{+} double hormone cells (arrows) appear in *Pdx1$^{m/m}$* mutant axolotls. Scale bar: 50 µm; Scale bar of magnified views: 10 µm.
